# Management and Care of Patients With Invasive Cervical Cancer: ASCO Resource-Stratified Guideline Rapid Recommendation Update

**DOI:** 10.1200/GO.22.00027

**Published:** 2022-03-04

**Authors:** Linus T. Chuang, Sarah Temin, Jonathan S. Berek

**Affiliations:** ^1^ Stanford University School of Medicine, Stanford, CA; ^2^ Danbury Hospital and Norwalk Hospital, Nuvance Health, Norwalk, CT; ^3^ American Society of Clinical Oncology, Alexandria, VA

## Abstract

ASCO Rapid Recommendations Updates highlight revisions to select ASCO guideline recommendations as a response to the emergence of new and practice-changing data. The rapid updates are supported by an evidence review and follow the guideline development processes outlined in the ASCO Guideline Methodology Manual. The goal of these articles is to disseminate updated recommendations, in a timely manner, to better inform health practitioners and the public on the best available cancer care options.

## BACKGROUND

In 2016, ASCO published a Resource-Stratified Guideline on the Management and Care of Women with Invasive Cervical Cancer.^1^ A recent publication^2^ constituted a strong signal for an update of the 2016 Invasive Cervical Cancer Resource-Stratified Guideline recommendations focused specifically on systemic therapy for patients with recurrent or metastatic cervical cancer in enhanced and maximal settings.

## METHODS

A targeted literature search was conducted to identify phase III clinical trials pertaining to the systemic therapy recommendations in this patient population. No additional randomized trials were identified. The original Expert Panel was reconvened to review the evidence from the KEYNOTE-826 trial and to approve the updated recommendation.

## EVIDENCE REVIEW

The KEYNOTE-826 investigators reported a first interim analysis of a double-blind, phase III randomized trial (617 patients) of pembrolizumab plus paclitaxel/platinum chemotherapy with or without bevacizumab compared with placebo plus chemotherapy with or without bevacizumab in patients with persistent, recurrent, or metastatic cervical cancer who had not received prior chemotherapy, with a median follow-up of 22 months.^2^ Patients with programmed death ligand 1 (PD-L1) ≥ 1 were 89% of each arm. Compared with a placebo/chemotherapy regimen, in all patients regardless of PDL-1 status, the progression-free survival (PFS) was significantly longer, 10.4 (95% CI, 9.1 to 12.1) versus 8.2 (95% CI, 6.4 to 8.4) months, with a hazard ratio (HR) of 0.65 (95% CI, 0.53 to 0.79; *P* < .001) in the pembrolizumab group. The overall survival (OS) was similarly longer in the pembrolizumab group (the coprimary end point) 24.4 versus 16.3-16.5 months (HR 0.67 [95% CI, 0.54 to 0.84; *P* < .001]). In patients with PD-L1 ≥ 1, PFS and OS were longer in the pembrolizumab group (PFS [HR 0.62 (95% CI, 0.50 to 0.77; *P* < .001)]).

Adverse event (AE) results were reported with the median treatment duration of 10 versus 7.7 months. Grade (Gr) ≥ 3 AEs (reported by ≥ 20% of patients) were numerically greater, 81.8% versus 75.1%, with intervention, but statistically similar (Table 1). Most common Gr ≥ 3 AEs were anemia (30.3% *v* 26.9%) and neutropenia (12.4% *v* 9.7%). Potentially immune-mediated AEs in the as-treated participants were greater with pembrolizumab (11.4% [Gr ≥ 3] *v* 2.9% [Gr 3-4]). In the as-treated participants analyzed by concomitant bevacizumab use, pembrolizumab plus bevacizumab had 83.7% Gr ≥ 3 AEs versus pembrolizumab alone 78.4%.

## 2016 RECOMMENDATION

Prior to these data's publication, the Invasive Cervical Cancer Resource-Stratified Guideline Panel published this recommendation in 2016 for patients with persistent, recurrent, or metastatic cervical cancer: Chemotherapy ± bevacizumab ± individualized radiation therapy and/or palliative care (Type of recommendation: evidence based; Evidence: high; Recommendation: strong). Other recommendations depend on previous radiation therapy and central versus noncentral disease (space precludes full reprinting; see 2016 guideline's Table 4).

## UPDATED RECOMMENDATION

The updated recommendation (plus the other 2016 options) for January 2022 is: clinicians may offer upfront pembrolizumab and chemotherapy with or without bevacizumab to eligible patients with persistent, recurrent, or metastatic cervical carcinoma (± individualized radiation therapy and/or palliative care) in enhanced and maximal settings (Type: evidence based, benefits outweigh harms; Evidence quality: high; Strength of recommendation: strong).

## DISCUSSION

Estimated OS and PFS were greater with pembrolizumab plus paclitaxel/platinum chemotherapy with or without bevacizumab versus a control with statistically significant difference at the time of this interim analysis (22-month follow-up). Although the results support use in all patients on the basis of intention to treat (ITT) analysis, investigators showed larger efficacy in the PD-L1 ≥ 1% participants. The subgroup analyses for both PFS and OS suggest that benefit may be less strong for patients with PD-L1 < 1% (HR 0.94).

The investigators found safety similar in both arms, with exceptions, for example, higher Gr 3 neutropenia and all Gr hypothyroidism with pembrolizumab (Table 1). With bevacizumab, higher AEs suggest higher toxicity, with potentially increased efficacy; the Panel encourages further research on its role. The investigators did not find significant problems with quality of life. The Panel recognizes that this regimen is not routinely available in resource-constrained settings and refers readers to the 2016 guidance.

**TABLE 1:**
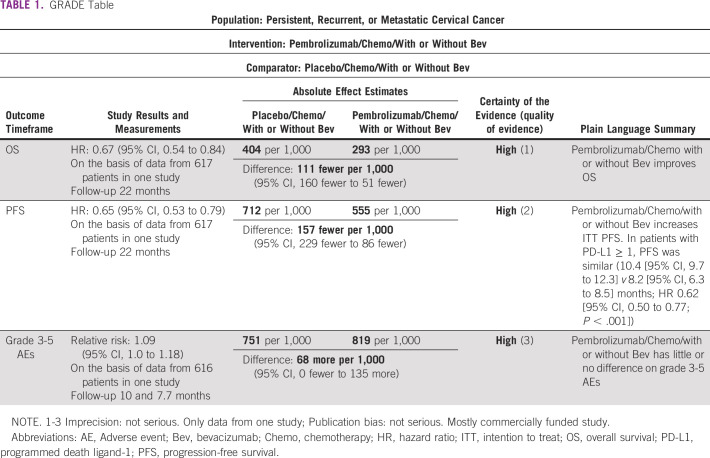
GRADE Table

## EMERGING EVIDENCE

The Expert Panel reviewed the single-arm innovaTV 204 trial and will evaluate future results of this and other trials in future full guideline updates per standard ASCO processes.

## GUIDELINE DISCLAIMER

The Clinical Practice Guidelines and Rapid Updates published herein are provided by the ASCO to assist providers in clinical decision making. The information herein should not be relied upon as being complete or accurate, nor should it be considered as inclusive of all proper treatments or methods of care or as a statement of the standard of care. With the rapid development of scientific knowledge, new evidence may emerge between the time information is developed and when it is published or read. The information is not continually updated and may not reflect the most recent evidence. The information addresses only the topics specifically identified therein and is not applicable to other interventions, diseases, or stages of diseases. This information does not mandate any particular course of medical care. Further, the information is not intended to substitute for the independent professional judgment of the treating provider, as the information does not account for individual variation among patients. Recommendations specify the level of confidence that the recommendation reflects the net effect of a given course of action. The use of words like “must,” “must not,” “should,” and “should not” indicates that a course of action is recommended or not recommended for either most or many patients, but there is latitude for the treating physician to select other courses of action in individual cases. In all cases, the selected course of action should be considered by the treating provider in the context of treating the individual patient. Use of the information is voluntary. ASCO does not endorse third party drugs, devices, services, or therapies used to diagnose, treat, monitor, manage, or alleviate health conditions. Any use of a brand or trade name is for identification purposes only. ASCO provides this information on an “as is” basis and makes no warranty, express or implied, regarding the information. ASCO specifically disclaims any warranties of merchantability or fitness for a particular use or purpose. ASCO assumes no responsibility for any injury or damage to persons or property arising out of or related to any use of this information, or for any errors or omissions.

## GUIDELINE AND CONFLICTS OF INTEREST

The Expert Panel was assembled in accordance with ASCO's Conflict of Interest Policy Implementation for Clinical Practice Guidelines (“Policy,” found at http://www.asco.org/rwc). All members of the Expert Panel completed ASCO's disclosure form, which requires disclosure of financial and other interests, including relationships with commercial entities that are reasonably likely to experience direct regulatory or commercial impact as a result of promulgation of the guideline. Categories for disclosure include employment; leadership; stock or other ownership; honoraria, consulting or advisory role; speaker's bureau; research funding; patents, royalties, other intellectual property; expert testimony; travel, accommodations, expenses; and other relationships. In accordance with the Policy, the majority of the members of the Expert Panel did not disclose any relationships constituting a conflict under the Policy.

## EDITOR'S NOTE

This ASCO Guideline Recommendation Update provides a recommendation update, with review and analysis of the relevant literature for the recommendation. Additional information, including links to patient information at www.cancer.net, is available at www.asco.org/resource-stratified-guidelines.
